# On the Valorization of Olive Oil Pomace: A Sustainable Approach for Methylene Blue Removal from Aqueous Media

**DOI:** 10.3390/polym16213055

**Published:** 2024-10-30

**Authors:** El Mokhtar Saoudi Hassani, Hugo Duarte, João Brás, Abdeslam Taleb, Mustapha Taleb, Zakia Rais, Alireza Eivazi, Magnus Norgren, Anabela Romano, Bruno Medronho

**Affiliations:** 1 Laboratory of Engineering Electrochemistry, Modeling, and Environment, Department of Chemistry, Faculty of Sciences Dhar Mahraz, Sidi Mohamed Ben Abdellah University, Fez 30000, Morocco; mustapha.taleb@usmba.ac.ma (M.T.); zakia.rais@usmba.ac.ma (Z.R.); 2 MED—Mediterranean Institute for Agriculture, Environment and Development, CHANGE–Global Change and Sustainability Institute, Faculdade de Ciências e Tecnologia, Universidade do Algarve, Campus de Gambelas, 8005-139 Faro, Portugal; jtbras@ualg.pt (J.B.); aromano@ualg.pt (A.R.); 3 Laboratory of Water and Environmental Engineering, Faculty of Sciences and Techniques of Mohammedia, Hassan II University of Casablanca, Mohammedia 28806, Morocco; talebabdeslam1@gmail.com; 4 Surface and Colloid Engineering, FSCN Research Center, Mid Sweden University, SE-851 70 Sundsvall, Sweden; alireza.eivazi@miun.se (A.E.); magnus.norgren@miun.se (M.N.)

**Keywords:** olive oil pomace, methylene blue, wastewater treatment, absorption kinetics, bio-based adsorbent

## Abstract

Currently, industrial water pollution represents a significant global challenge, with the potential to adversely impact human health and the integrity of ecosystems. The continuous increase in global consumption has resulted in an exponential rise in the use of dyes, which have become one of the major water pollutants, causing significant environmental impacts. In order to address these concerns, a number of wastewater treatment methods have been developed, with a particular focus on physicochemical approaches, such as adsorption. The objective of this study is to investigate the potential of a bio-based material derived from olive oil pomace (OOP) as an environmentally friendly bio-adsorbent for the removal of methylene blue (MB), a cationic dye commonly found in textile effluents. The biobased material was initially characterized by determining the point of zero charge (pHpzc) and using scanning electron microscopy (SEM), X-ray diffraction (XRD), and Fourier transform infrared spectroscopy (FTIR). Subsequently, a comprehensive analysis was conducted, evaluating the impact of specific physicochemical parameters on MB adsorption, which included a thorough examination of the kinetic and thermodynamic aspects. The adsorption process was characterized using Langmuir, Freundlich, Brunauer-Emmett-Teller (BET), and Dubinin Radushkevich (D-R) isotherms. The results suggest that the equilibrium of adsorption is achieved within ca. 200 min, following pseudo-second-order kinetics. The optimal conditions, including adsorbent mass, temperature, bulk pH, and dye concentration, yielded a maximum adsorption capacity of ca. 93% (i.e., 428 mg g^−1^) for a pomace concentration of 450 mg L^−1^. The results suggest a monolayer adsorption process with preferential electrostatic interactions between the dye and the pomace adsorbent. This is supported by the application of Langmuir, BET, Freundlich, and D-R isotherm models. The thermodynamic analysis indicates that the adsorption process is spontaneous and exothermic. This work presents a sustainable solution for mitigating MB contamination in wastewater streams while simultaneously valorizing OOP, an agricultural by-product that presents risks to human health and the environment. In conclusion, this approach offers an innovative ecological alternative to synthetic adsorbents.

## 1. Introduction

The availability of water resources is a fundamental determinant of socio-economic processes, irrespective of the stage of societal development [1]. The advent of scientific and technological advancement has given rise to a plethora of novel challenges and opportunities. However, it has also precipitated a range of serious environmental concerns, including pollution of the atmosphere, land, and water-based ecosystems [2]. This has already resulted in irreversible damage to numerous natural resources, thereby threatening the delicate ecological equilibrium that is crucial for supporting life on Earth [3]. It is estimated that 34% of the global population will face challenges associated with the lack of access to clean and safe water suitable for drinking and other domestic purposes [4]. The textile industry is among the sectors that contribute the most to water pollution. A significant fraction of this pollution is caused by the release of colored effluents containing numerous toxic dyes into diverse water bodies without proper treatment [5]. These substances have been identified as a long-standing environmental concern due to their toxic, carcinogenic, mutagenic, and teratogenic characteristics [6]. The lack and/or inadequacy of treatment systems in this industry results in the accumulation of these pollutants within the water cycle [7].

Despite the implementation of a range of programs at the international and national levels that aimed at addressing the global water crisis caused by indiscriminate discharges and setting the world’s trajectory towards sustainable development and equitable water access for all, the issue of surface water contamination continues to represent a significant global concern [8].

Similarly, the decolorization of textile effluents has been the focus of numerous studies, as has the treatment of other hazardous organic substances. Several physicochemical processes have been the subject of extensive investigation with a view to determining their efficacy in decolorizing aqueous media. These include coagulation-flocculation [9,10], oxidation [11], and membrane filtration [12,13], which have been demonstrated to be highly efficient in this regard. In contrast, biological-based treatment processes are seldom utilized for the remediation of water contaminated by dyes, primarily due to their low biodegradability [14]. Furthermore, many of these techniques continue to exhibit significant limitations, including the excessive use of chemicals, the accumulation of concentrated sludge, which presents challenging disposal issues, particularly when dealing with high-flow effluents, elevated treatment costs, and high energy consumption [15]. Among all the various strategies available, adsorption-based methods are particularly effective for the removal of dyes from wastewater. They offer several advantages, including ease of operation, sustainability, cost-effectiveness, and widespread availability, making them a highly attractive option for wastewater treatment [16,17]. Extraction of dyes from aqueous solutions using a variety of materials, with a particular emphasis on activated carbon [18,19], has been the subject of extensive research. In recent years, a number of adsorbents have been proposed for the removal of dyes, including clay [20], layered double hydroxides [21], metal oxides [22], modified natural goethite [23], and graphene oxide [24]. However, these materials are not cost-effective, are derived from non-renewable resources, and exhibit a very limited recyclability and reusability. It is therefore imperative to investigate alternative, cost-effective, efficient, and eco-friendly options to traditional adsorbents [25].

In this regard, recent studies have highlighted the potential of bio-based materials for wastewater treatment and dye removal. These bio-based materials, including agricultural waste, biomass, and biopolymers, have emerged as promising solutions due to their abundance, low cost, and biodegradability. For example, studies have demonstrated the efficacy of coffee grounds [26], Aleppo pine branches [27], walnut shells [28], fruit peels [29], date pits [30], wheat shells [31], mushrooms [32], chitosan [33], natural zeolite [34] and olive stone [35] in the removal of dyes from wastewater. These bio-based materials are not only sustainable and environmentally friendly, as they also offer comparable or, in some cases, even superior adsorption performance to that of conventional materials. This makes their use particularly attractive for large-scale applications.

The olive oil pomace (OOP), a significant byproduct of olive oil production in the Mediterranean, can be regarded as an attractive bio-based material. The olive oil market has experienced a notable expansion over the past two decades, with Morocco emerging as a prominent global player. In 2020, Morocco produced approximately 160,000 metric tons of olive oil, ranking it among the top five global producers. Olive trees are cultivated across Morocco, with an area of over 1.2 million hectares [36]. The remarkable adaptability of olive trees to a variety of bioclimatic zones, from humid to arid and semi-arid, makes them an invaluable asset in the socio-economic development of both national and regional agricultural sectors. In addition to its strategic value in combating erosion and revitalizing underperforming agricultural land, olive cultivation also plays a role in stabilizing populations in marginal areas. It is estimated that approximately 75% of olive farms are of a relatively small scale, which helps to maintain rural employment and prevent the exodus of the rural population. The olive oil sector is not only fundamental to the sustainability of agriculture, it also makes a significant contribution to regional economies, stimulating growth and prosperity [37].

As previously stated, the production of olive oil generates a considerable quantity of by-products, primarily OOP. The production of one tonne of processed olives results in the generation of approximately 400 kg of OOP [38], which equates to an estimated 640,000 metric tons of pomace produced annually in Morocco. Presently, these by-products are frequently inadequately managed, resulting in environmental issues. Nevertheless, there are indications that initiatives are emerging with the objective of valorizing this waste. For instance, pomace can be employed as a biofuel, an organic soil enhancer, or an adsorbent agent for wastewater treatment. The valorization of these by-products has the potential to mitigate environmental impact while simultaneously creating new economic opportunities.

The objective of this study is to evaluate whether the OOP can be employed as a natural adsorbent, thereby providing a sustainable alternative to synthetic adsorbents. Among the numerous synthetic dyes (adsorbates) that have been developed, methylene blue (MB) has become a prominent and increasingly utilized compound since its first synthesis in 1877 [39]. Its extensive use in a variety of industries, including textiles, pharmaceuticals, and paper production, can be attributed to its high solubility in water and vibrant coloration [40,41]. Given its persistence and prevalence in water effluents, even low concentrations of MB can have significant adverse effects, contributing to 20% of global wastewater pollution [42,43]. MB has been identified as a significant environmental contaminant with the potential to cause irreversible harm to aquatic organisms [43]. It is frequently discharged into water bodies without adequate treatment, resulting in pollution that is challenging to remediate due to the dye’s chemical stability [44]. When released into aquatic ecosystems, it can have a detrimental impact on marine life due to its toxicity and the way it hinders light penetration, which in turn affects photosynthetic processes [45]. The release of aromatic amines from MB in textile effluents represents a significant environmental and human health concern [46]. Consequently, the scientific community has been focusing on the development of affordable and effective long-term solutions for the removal of MB from water resources [47,48].

The objective of this study is to evaluate the capacity of the Moroccan OOP to remove MB. The pomace was subjected to a detailed characterization in order to ascertain its microstructural features, crystallinity, and functional groups. Furthermore, the adsorption conditions, including the initial concentration of the dye, the mass of the adsorbent, the temperature, and the pH, were optimized. The results obtained were subjected to modeling using Langmuir, Freundlich, BET, and D-R isotherms to elucidate the adsorption mechanism and assess kinetic and thermodynamic parameters.

## 2. Materials and Methods

### 2.1. Materials

Methylene blue (97%) was supplied by VWR Chemicals, while HCL (37%) was purchased from Sigma Aldrich (St. Louis, MO, USA). Sodium hydroxide (98%) and ethyl acetate (99.5%) were both supplied by Panreac (Castellar del Vallès, Spain). The OOP, a dried blackish-brown solid residue, was collected from a natural evaporation storage basin located at Meknassa ben Ali, 8 km from the town of Taza in Morocco.

### 2.2. Pre-Treatment of the Olive Oil Pomace

**Figure 1:**
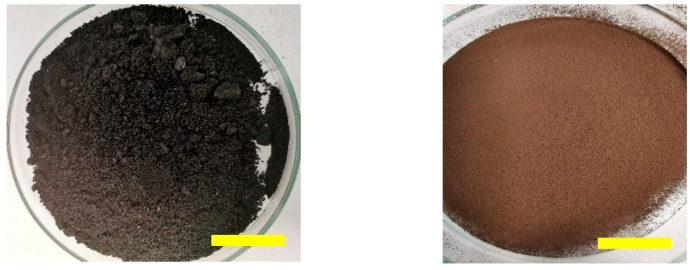
OOP after crushing (**left**) and OOP after defatting and sieving (**right**). The scale bar represents 2 cm.

The collected solid OOP was pulverized with a crusher (Laarmann LMC100D), and the resultant material was subsequently milled using a high-powered mill (VEVOR XZ-8B). The obtained powder was defatted with five solid-liquid extractions using ethyl acetate 1:2 (*w*/*v*). The defatted material was then dried in an oven at 30 °C for 24 h. Finally, the material was sieved through a 0.2 mm sieve to obtain a fine powder. In Figure 1, photographs of the initial crushed OOP (left image) and defatted and sieved OOP (right image) are shown.

### 2.3. Methods

#### 2.3.1. SEM

Field emission scanning electron microscopy (FE-SEM) imaging of OOP samples was carried out using a TESCAN MAIA3 electron microscope in secondary electron (SE) mode. The accelerating voltage was 5 kV, and the work distance was set to 5–6 mm. Before image acquisition, the samples were coated with 6 nm iridium using a Quorum Q150T ES.

#### 2.3.2. XRD

X-ray diffraction (XRD) was performed at room temperature using a Bruker D2 Phaser diffractometer with Cu Kα radiation (wavelength 1.54 Å) at 30 kV and 10 mA, in θ–2θ geometry. The increment was fixed at 0.1°, and the 2ϴ range was from 5° to 80°. The samples were placed on a silicon single crystal specially cut to provide a low background free from any interfering diffraction peaks.

#### 2.3.3. FTIR

The IR analyses were conducted using a Bruker Tensor 27 (Billerica, MA, USA). Samples from the OOP powder were prepared by pressing them with KBr to form pellets. These pellets were then analyzed over the range of 600–4000 cm^−1^, with 25 scans and a resolution of 4 cm^−1^, at room temperature.

#### 2.3.4. Point of Zero Charge (pHpzc)

The point of zero charge (pH_pzc_) was determined using the solid addition method. In this method [25,49], solutions consisting of 5 g of biobased material in 150 mL of 0.05 M NaCl were stirred for 48 h. The initial pH (pH_i_) of the solutions was adjusted by adding either 0.1 M HCl or KOH. After stirring, the final pH (pH_f_) of the supernatants was measured after settling. The difference between final and initial pH values (ΔpH = pH_f_ − pH_i_) was then plotted against pH_i_. The pH_pzc_ was identified as the point of intersection of the resulting curve with ΔpH = 0.

### 2.4. MB Adsorption

#### 2.4.1. Adsorption Kinetics

The kinetic study was conducted using a series of suspensions composed of 0.1 g of OOP in 100 mL of MB aqueous solutions, with concentrations ranging from 5 to 460 mg L^−1^, at pH 7 and room temperature (c.a. 20 °C). The mixtures were placed in an orbital shaker (Buhler Universal Shaker KS 15A) and subjected to continuous agitation of 300 rpm. Aliquots were periodically collected, subjected to centrifugation of 12,000 rpm (Mikro Hettic 200, Tuttlingen, Germany), and then analyzed in a UV-Vis spectrometer (T70+ UV/VIS Spectrometer, PG Instruments Ltd., Lutterworth, UK) by measuring the respective absorbance at 664 nm (i.e., absorbance maximum of MB). The adsorption capacity of OOP-based residue was determined using Equation (1):

(1) $$Q_{t}=\frac{\left(C_{0}-C_{e}\right)\cdot V}{m}$$

where *C*_0_ (mg L^−1^) represents the initial dye concentration, *C_e_* (mg L^−1^) denotes the dye concentration at equilibrium conditions, *V* (L) is the dye volume, and *m* (g) indicates the mass of adsorbent in the solution [49].

Data was further modeled considering pseudo-first order (Equation (2)) and pseudo-second order (Equation (3)) kinetics [50].

(2) $$\ln\left(q_{e}-q_{t}\right)=\ln\left(q_{e}\right)-k_{1}\cdot t$$

(3) $$\frac{t}{q_{t}}=\frac{1}{k_{2}{\cdot q_{e}}^{2}}+\frac{t}{q_{t}}$$

where *q_e_* and *q_t_* represent the mass of adsorbed material (mg g^−1^) at equilibrium and at a given time *t*, respectively. The parameter *k*_1_ (min^−1^) corresponds to the pseudo-first-order kinetic constant, while *k*_2_ denotes the pseudo-second-order kinetic constant (g mg min^−1^).

The initial rate of adsorption, *h*, when *t* = 0 is defined by Equation (4):

(4) $$h=k_{2}\cdot q_{e}^{2}$$

where *q_e_*, *h*, and *k*_2_ are calculated based on the slope and intercept of the *t*/*q_t_* linear curve as a function of time.

#### 2.4.2. Adsorption Equilibrium

The adsorption isotherms were calculated in equilibrium conditions, employing the same parameters as in the adsorption kinetics experiments. The solutions were stirred at 300 rpm until equilibrium was reached at room temperature (20 ± 2 °C), with the pH of the medium consistently maintained at 7. Additional optimization essays were carried out by varying a given parameter in the study, keeping the other previous optimal conditions fixed, except in the cases where the optimal conditions had not yet been determined, and thus standard conditions were used (e.g., neutral pH and room temperature). The isothermal models considered include Langmuir, Freundlich, BET, and Dubinin Radushkevich (D-R), as these models best capture the organic dye adsorption mechanisms [51,52].

In the Langmuir model, the adsorption relies on the concept of an adsorbate monolayer formation on the surface of the adsorbent, and it is generally represented by Equation (5) [53]:

(5) $$q_{e}=\frac{q_{m}\cdot K_{L}\cdot C_{e}}{1+K_{L}\cdot C_{e}}$$

where *q_m_* (mg g^−1^) represents the maximum adsorption capacity, *K_L_* (L mg^−1^) represents the Langmuir constant associated with the affinity of binding sites and adsorption energy, and *C_e_* is the concentration of the dye in solution at equilibrium (mg L^−1^). Plotting *C_e_*/*q_e_* as a function of *C_e_*, allows determining *q_m_* and *K_L_*. The key features of the Langmuir isotherm can be summarized in the separation factor (*R_L_*) [54], which can be computed using Equation (6):

(6) $${\;R}_{L}=\frac{1}{1+K_{L}\cdot \;C_{0}}$$

With *C*_0_ being the starting concentration of absorbate (mg L^−1^). The analysis of *R_L_* suggests the adsorption type (Table 1).

In turn, the Freundlich isotherm assumes that adsorption occurs on a heterogeneous surface through a multi-layer adsorption process and that the quantity adsorbed increases proportionally to the concentration. It is defined by Equation (7) [55]:

(7) $$q_{e}=K_{F}\cdot {C_{e}}^{\frac{1}{n}}$$

With *K_F_* [(mg g^−1^)(L mg^−1^)^1/n^] being a distribution coefficient/constant indicative of the adsorption capacity and n being a dimensionless correction factor associated with the intensity of adsorption [56]. The 1/*n* parameter indicates the deviation of the adsorption isotherm from linearity. When 1/*n* = 1, the adsorption process is linear, thus suggesting homogeneity of sites and an absence of interactions between the adsorbed species. When 1/*n* < 1, the adsorption process is considered favorable and is followed by an increase in the retention capacity and the emergence of numerous new adsorption sites. On the other hand, when 1/*n* > 1, the adsorption process is unfavorable, and the adsorption chains weaken, leading to a reduction in adsorption capacity [56].

The D-R isotherm model is usually used to express an adsorption mechanism with a Gaussian energy distribution into heterogeneous surfaces [57].

(8) $$q_{e}=q_{m}-e^{-K_{D}\varepsilon^{2}}$$

*K_D_* (mol^2^ kJ^−2^) is the D-R model constant, and *ε* (kJ mol^−1^) is the adsorption potential, which can be calculated as follows [57]:

(9) $$\varepsilon =R\cdot T\cdot ln\frac{C_{s}}{C_{e}}$$

where *C_s_* (mg L^−1^) is the solubility of the adsorbate and *C_e_* (mg L^−1^) is the equilibrium concentration of adsorbate remaining in solution. The mean free energy, *E* (kJ mol^−1^), can be calculated using the following equation:

(10) $$E=\frac{1}{\sqrt{2K_{D}}}$$

This model is commonly used to determine whether the adsorption process is mainly physical (*E* < 16 kJ mol^−1^), involving interactions such as electrostatic forces, hydrogen bonding, or pi-pi interactions, or chemical (*E* > 16 kJ mol^−1^), involving the formation of novel chemical bonds [58].

The BET model is a theoretical multi-layer physical adsorption formalism, assuming homogenous adsorption where the adsorption energy of the first layer differs from the other layers. The model also takes a pseudo-steady state into account, a dynamic equilibrium where the rates of adsorption and desorption are equivalent [59].

(11) $$q_{e}=\frac{q_{m}\cdot K_{L}\cdot C_{e}}{\left(1-K_{S}\cdot C_{e}\right)[1+\left(K_{L}-K_{S}\right)C_{e}]}$$

*K_L_* (L mg^−1^) is the monolayer adsorption equilibrium constant, and *K_S_* is the multilayer adsorption equilibrium constant (L mg^−1^). Note that when *K_L_* = 0, the BET isotherm simplifies to the Langmuir model, representing a monolayer adsorption process, and *K_L_* becomes the Langmuir constant.

Lastly, the thermodynamics of the adsorption process, specifically the Gibbs free energy (ΔG°), the enthalpy (ΔH°), and entropy (ΔS°), were assessed via the following equations [60]:

(12) $$K_{d}=\frac{q_{e}}{C_{e}}$$

(13) $$\Delta G{}^{\circ}=-R\cdot T\cdot lnK_{d}$$

(14) $$lnK_{d}=\left(\frac{\Delta S{}^{\circ}}{R}\right)-\left(\frac{\Delta H{}^{\circ}}{R}\right)\frac{1}{T}$$

where *K_d_* is the distribution constant deduced from the adsorption capacity *q_e_* of the pomace adsorbent and *C_e_*.

### 2.5. Methylene Blue Desorption

Ethanol was used as a desorption agent to remove MB adsorbed on the OOP. The desorption protocol was carried out by immersing 25 mg of OOP, loaded with different concentrations of MB at pH levels of 7 and 10, in a volume of 10 mL of ethanol solution for 6 h at room temperature. After this period, the mixture was filtered to separate the OOP from the desorbed solution, thus allowing for the quantification of the recovered MB using spectrophotometry and determining the desorption efficiency. The cycles of MB removal from the OOP involved repeating this desorption process multiple times. After each adsorption cycle, where the OOP was exposed to a MB solution, it underwent desorption with ethanol. This approach allows for evaluating the effectiveness of the OOP as an adsorbent and analyzing its regeneration capacity for repeated applications in the treatment of colored effluents.

## 3. Results and Discussion

### 3.1. Characterization of Olive Oil Pomace

**Figure 2:**
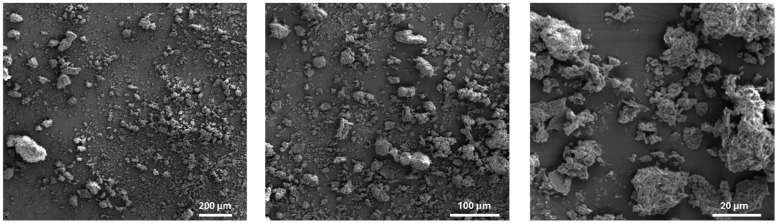
SEM micrographs of the OOP particles.

Following the sieving and defatting of the OOP, an assessment of the material’s morphological features was conducted via scanning electron microscopy (SEM) at varying magnifications (Figure 2). The electron micrographs revealed that the material is composed of condensed agglomerates. The formation of these aggregates indicates the presence of a complex and interconnected structure, which in turn gives rise to a porous network of considerable interfacial area. This network may potentially exhibit effective adsorbent properties. The porous structure provides numerous sites for the adsorption of dyes [61].

**Figure 3:**
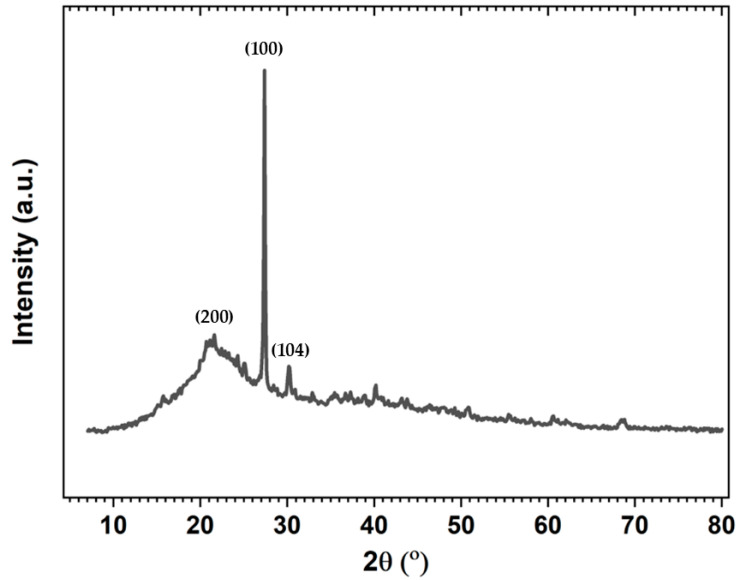
XRD diffraction pattern of defatted and sieved OOP.

In its raw state, the OOP is predominantly composed of water (80–83%), organic substances (15–18%), and inorganic substances (2%) [62]. As illustrated in Figure 3, the XRD diffraction pattern exhibits prominent peaks at approximately 2θ = 22°, 27°, and 30.5°, which correspond to the characteristic Bragg diffractions of lignocellulosic materials. In particular, the peak at 2θ = 22°, which corresponds to the diffraction of (200) crystallographic plane reflection, is indicative of an amorphous or poorly crystalline phase, which is typical of cellulose type I allomorph. The sharp and intense peak around 2θ = 27° and 30° suggests a crystalline phase with significant ordering. It is also possible that the olive stone contributes to this observed pattern. It is notable that the reflection at 2θ = 22° is broader in comparison to the other peaks, which lends support to the presence of amorphous material, a common feature of organic materials such as lignin, cellulose, and hemicellulose [35,63]. Conversely, the peak at 2θ = 27° is often attributed to quartz (SiO_2_) and corresponds to the (100) crystallographic plane reflection, while the peak at 2θ = 30° is attributed to calcite (CaCO_3_) and corresponds to the (104) crystallographic plane reflection. Further smaller peaks are noticed and may correspond to sylvite (KCl), muscovite [KAl_2_(AlSi_3_O_10_)(OH)_2_], and haematite (Fe_2_O_3_). The presence of these minerals can be attributed to soil residues or as a natural mineral component, as has been previously observed in other studies [64,65,66].

**Figure 4:**
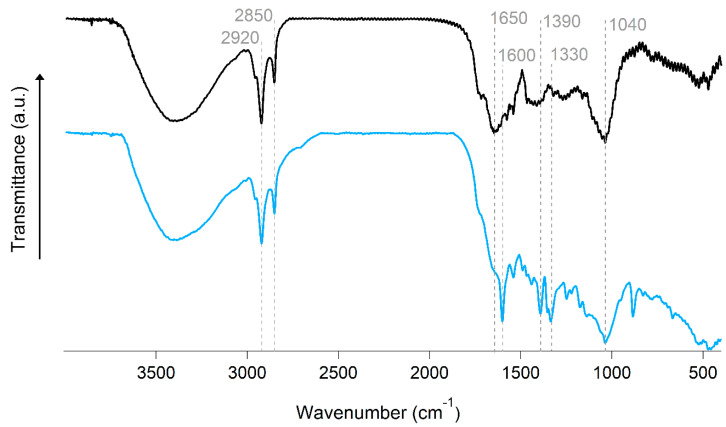
FTIR spectra of OOP-biobased material, before (black) and after (blue) MB adsorption.

The OOP was further characterized by FTIR (Figure 4). A broad absorption peak at 3400 cm^−1^ is attributed to hydroxyl (-OH) vibration modes, which are likely associated with alcohol, phenol, or carboxyl functional groups [67]. Two bands at 2920 cm^1^ and 2850 cm^1^ are assigned to the stretching of methyl (-CH_3_) and methylene groups (-CH_2_), respectively [68]. The peak at 1650 cm^−1^ is ascribed to the carboxyl (-C=O) vibration in phenolic components [69], while the prominent band at 1040 cm^−1^ is attributed to C-O stretching vibrations in alcohols [70]. Following the adsorption of MB, new bands indicative of MB emerge at 1600, 1390, and 1330 cm^−1,^ which are characteristic vibrational modes for MB [71]. Furthermore, the FTIR data indicate the presence of a considerable number of oxygenated groups on the OOP biobased material, which is anticipated to facilitate favorable interactions, predominantly hydrogen bonding with the MB dye, thereby enhancing its successful adsorption [72].

Following the morphological and chemical characterization of the OOP, the pH_pzc_ was determined with the objective of inferring the impact of pH on the OOP surface charge density. The pH_pzc_ was identified at pH 5.4, which represents a critical threshold at which the surface charge of the biobased material becomes electrically neutral [73]. This indicates that, when the solution pH is greater than 5.4, the deprotonation of the OOP functional groups should result in the formation of a negatively charged surface [74]. A comprehensive understanding of the surface charge features dependence on pH provides valuable insights into its potential applications, particularly in scenarios where interactions with charged species play a pivotal role, as in the case of dye removal [75,76]. This emphasizes the significance of pH as a crucial factor influencing the surface charge of the biobased material and, as it will be further elucidated in the subsequent sections, in relation to its adsorption capacity.

### 3.2. MB Adsorption

#### 3.2.1. Adsorption Kinetics

**Figure 5:**
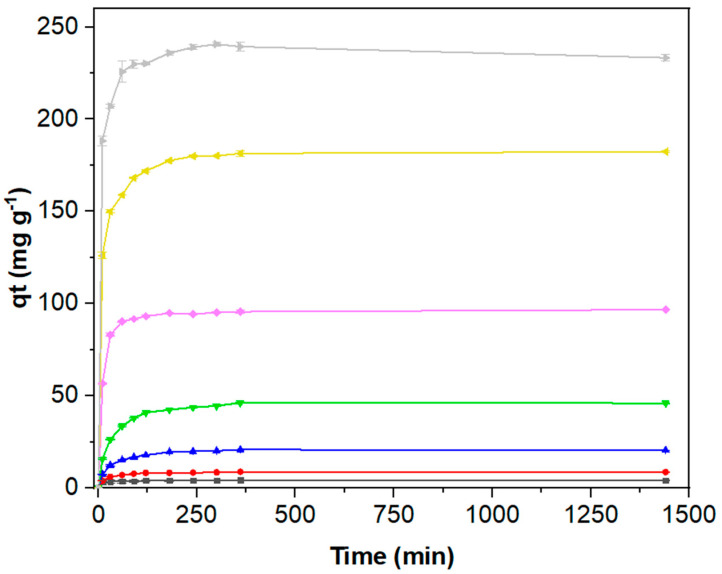
Adsorption kinetics of MB on OOP at pH 7 and at a temperature of 20 °C. The adsorbent mass was kept at 1 g L^−1^ while the MB concentration varied from 5 to 460 mg L^−1^; 5 (black), 10 (red), 20 (blue), 50 (green), 100 (pink), 200 (yellow), and 460 mg L^−1^ (grey).

Once the pHpzc of the OOP had been established, the adsorption kinetics assays were performed as described in the experimental section. Figure 5 illustrates a discernible pattern in the removal of MB over time. In the initial stage, spanning the first 50 minutes, and regardless of the MB concentration, there is a rapid increase in the amount of dye adsorbed. During this phase, the dye is expected to predominantly adsorb onto readily accessible sites of the pomace [77]. Conversely, the second stage is distinguished by a notable deceleration in the adsorption rate. This deceleration suggests a transition in the adsorption process, whereby the diffusion is likely occurring towards non-saturated and less accessible adsorption sites. As the number of available binding sites increases, competition for these limited sites begins. This phase persists until an equilibrium plateau in adsorption is achieved, marking the onset of the third stage. The equilibrium stage is reached for all concentrations after ca. 200 min, although the adsorbed amount of dye was found to increase with the initial concentration of dye. This suggests an increase in the driving force for mass transfer, whereby higher initial concentrations result in a greater concentration gradient between the adsorbate and adsorbent molecules, facilitating their diffusion from areas of high chemical potential to areas of low chemical potential within physical systems. Such a gradient can act as a driving force, enhancing the rate at which the dye molecules diffuse and adhere to the adsorbent surface [77,78].

As previously stated, the observed kinetic profiles illustrate the dynamic nature of the adsorption process, emphasizing the existence of distinct phases associated with the accessibility of adsorption sites. These kinetic profiles can then be fitted with suitable mathematical models. The pseudo-first order (PFO) and pseudo-second order (PSO) kinetic models were evaluated using the formalism described in Equations (2) and (3). The values of adsorption rate constants (*k*_1_, *k*_2_), initial velocities (*h*), and equilibrium adsorption capacities (*q_e_*) are summarized in Table 2. As can be observed, the theoretically calculated adsorbed quantities (q_the_) obtained from the PFO modeling are notably low in comparison to the experimentally determined quantities, resulting in a poor linear regression correlation coefficient (R^2^).

**Figure 6:**
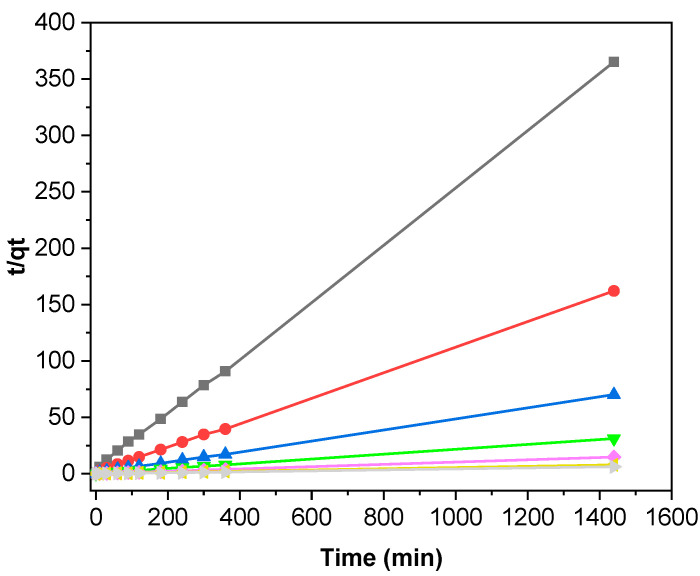
Application of the PSO model in the adsorption OOP for different MB concentrations at 20 °C, pH = 7, and an adsorbent mass of 1 g L^−1^: 5 (black), 10 (red), 20 (blue), 50 (green), 100 (pink), 200 (yellow), and 460 mg L^−1^ (grey).

These results suggest that the adsorption of MB on the OOP deviates from a controlled diffusion process, as it does not follow a PFO process. Conversely, the data could be perfectly fitted with a PSO model, as illustrated in Figure 6. Moreover, the R^2^ values for the PSO model exceed those for the PFO model, approaching unity, thereby indicating a robust fit of the PSO model. It is noteworthy that the q_the_ values are in close alignment with the experimentally determined values, which serves to demonstrate remarkable consistency and reinforces the validity of the PSO model in elucidating the adsorption kinetic process, irrespective of the dye concentration in the solution.

#### 3.2.2. Adsorption Isotherms

The adsorption of MB on the OOP was further investigated through the application of isothermal models, as outlined in the experimental section (i.e., Langmuir, Freundlich, BET, and D-R). The equilibrium parameters and constant values associated with the four isotherms were determined and compiled in Table 3 by applying the aforementioned mathematical models to the adsorption equilibrium data.

The Langmuir model was employed to extract the requisite parameters, which enabled the determination of the *R_L_* to be established at a value lower than 1 for each concentration of dye, varying from 0.99 at 20 mg L^−1^ MB, progressively decreasing to 0.84 when at 460 mg L^−1^ MB. As documented in the literature, this should result in a favorable adsorption process [79]. In addition to the obtained R^2^ value, the Langmuir model appears to be an appropriate model for describing the adsorption process as a monolayer adsorption [80].

The calculation of 1/*n*, derived from the slope of the Freundlich isotherm curve (Table 3), yielded a value below 1, indicating a favorable MB adsorption on the pomace surface [81].

In order to account for the possibility that multilayer formation processes could contribute to the adsorption mechanism, the BET isotherm was applied. The optimal fit yielded a *K_L_* = 0 L mg^−1^, where the BET isotherm is reduced to the Langmuir equation, thus supporting a favorable monolayer formation process [52,59]. Furthermore, the D-R isotherm indicated that the mean value of the calculated free energy is ca. 10 kJ mol^−1^, suggesting a physical-driven adsorption process mainly governed by interactions such as electrostatic, hydrogen bonding, and pi-pi interactions [58]. Moreover, as demonstrated previously through XRD, the OOP contains several minerals, including quartz, muscovite, and hematite. These minerals are well-known adsorbents that can form multiple bonds with cationic dyes, such as MB. This results in low enthalpy and Gibbs free energy variations, as well as low adsorption energy [82]. Therefore, it can be concluded that the adsorption of MB onto the OOP is not solely determined by the physical interactions associated with its cellulose- and lignin-rich composition. In fact, the constitution of OOP is well known, with its main components being cellulose (14–21%), hemicellulose (19–30%), and lignin (36–42%) [83]. Additionally, the presence of various minerals may contribute to the adsorption process through the formation of ionic interactions [74].

It is also assumed that the heat of adsorption of all the molecules in the layer falls linearly with surface coverage due to the interaction between the adsorbent and adsorbate. Additionally, the adsorption is characterized by a uniform distribution of the bonding energies, up to some maximum binding energy [82,84]. Furthermore, the thermodynamic parameter results, which are presented in Table 4, provide crucial insights into the nature of the adsorption process. The standard Gibbs free energy (ΔG°) values, which range from −355.47 to −1177.1 kJ mol^−1^, indicate that the adsorption of MB onto OOP is spontaneous at all temperatures studied. Moreover, the reduction in ΔG° with increased temperature indicates that adsorption is more favorable adsorption at higher temperatures [85]. Conversely, the standard enthalpy (ΔH°) is positive at 5496.21 kJ mol^−1^, indicating that the process is endothermic, whereby heat is absorbed from the environment during adsorption. Additionally, the positive value of the standard entropy (ΔS° = 36 J mol^−1^) demonstrates the affinity of OOP for MB and the occurrence of structural changes in the adsorbent, indicating the presence of random interactions at the solid-liquid interface [86].

#### 3.2.3. Optimization of the Adsorption Process

**Figure 7:**
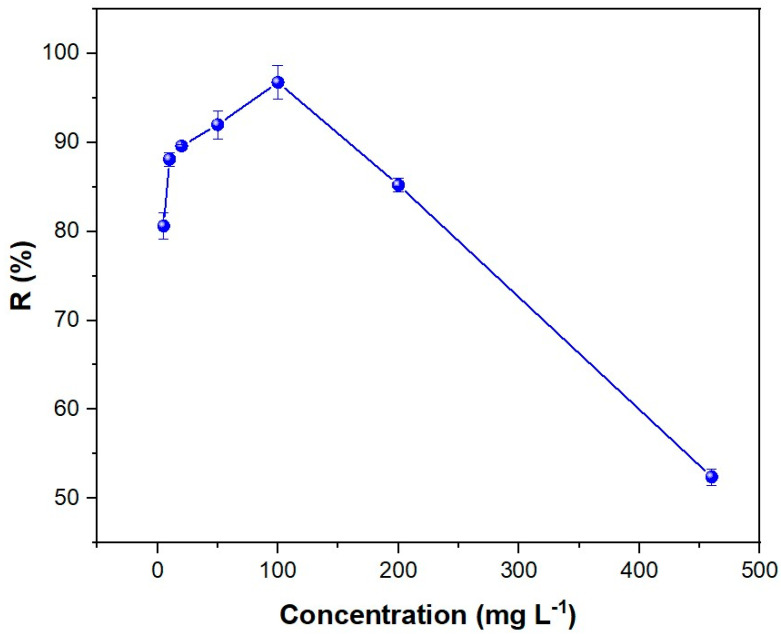
Effect of dye concentration on the adsorption efficiency of OOP. The adsorbent mass was 1 g L^−1,^ and the system was stirred for 200 min at pH 7 and 20 °C.

The impact of the initial dye concentration on the adsorption process was examined by conducting a systematic variation in concentration from 5 to 460 mg L^−1^ (Figure 7). The *absorption efficiency* can be estimated from the following equation:

(15) $$Adsorption\;efficiency\;(\%)=\frac{C_{0}-C_{eq}}{C_{0}}\cdot 100$$

where *C*_0_ is the initial dye concentration (i.e., 460 mg L^−1^) and *C_eq_* is the dye concentration at equilibrium time. The relationship between solution decolorization and dye concentration demonstrates that the maximum adsorption efficiency (96.3%) is achieved at a dye concentration of 100 mg L^−1^. It is anticipated that the removal yield of MB will decline or, at best, remain constant as the concentration increases, given that a greater number of adsorbate molecules will be available for adsorption. Accordingly, the adsorbent capacity should diminish as the number of available binding sites declines or remain constant until reaching full occupation in equilibrium [35]. It is noteworthy that the removal yield increases with the initial concentration before reaching the optimal level. One potential explanation for this phenomenon is the increase in the driving force for mass transfer as the concentration of the adsorbate rises, resulting from a greater concentration gradient between the adsorbate and the adsorbent [78]. This is contingent upon the availability of sufficient available active sites for binding. At concentrations above the optimal level, the removal yield declines due to the saturation of the active sites on OOP [87]. These factors have been previously described in detail in the kinetic study (Section 3.2.).

**Figure 8:**
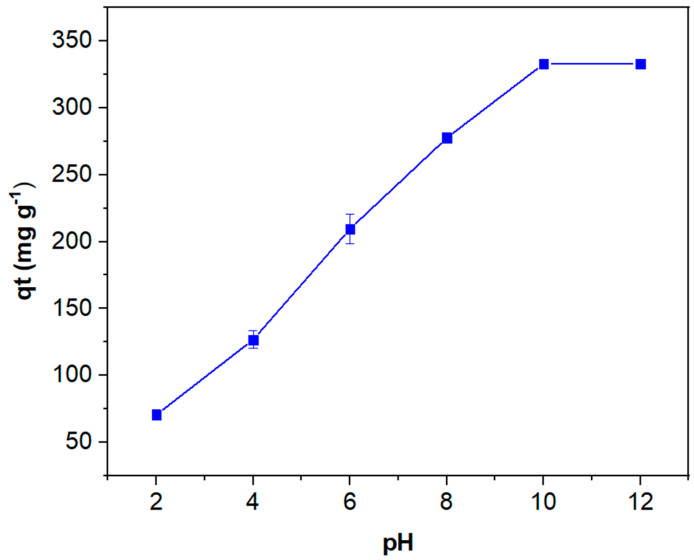
Effect of solution pH on the adsorption efficiency of OOP (dye concentration of 460 mg L^−1^; adsorbent mass of 1 g L^−1^, after stirring the sample for 200 min at 20 °C).

Another important parameter to evaluate is pH, as both the biobased material and the dye are susceptible to its effects. The influence of pH was evaluated over the range 2 to 10, with the dye concentration held constant at 460 mg L^−1^. As illustrated in Figure 8, at an acidic pH of 2, the adsorption efficiency of MB reaches a minimum, with a corresponding adsorption quantity of ca. 70 mg g^−1^. At this low pH, the pomace biobased material exhibits the highest positive charge density due to the protonation of functional groups on its surface. This results in a reduced capacity for electrostatic interaction with positive species, thereby hindering the adsorption of the cationic MB dye. Moreover, the high concentration of H^+^ ions present in the solution competes with the MB molecules, thereby reducing their adsorption capacity [88]. Conversely, at a pH above 5.4, the biobased material is negatively charged, thus favoring the electrostatic interaction with the cationic dye [89]. This is clearly demonstrated by the significant increase in adsorption observed as pH rises, with the highest level of adsorbed MB (ca. 333 mg g^−1^) being reached at pH 10.

**Figure 9:**
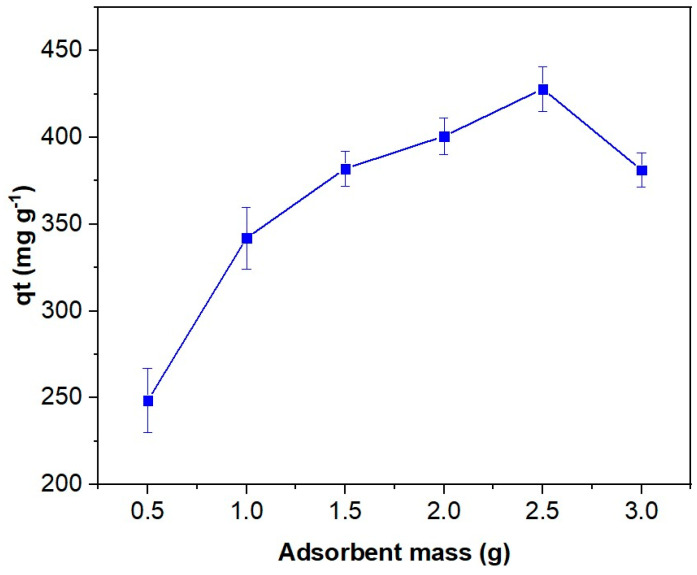
Effect of adsorbent concentration on the adsorption efficiency of MB (460 mg L^−1^) by the OOP after stirring the samples for 200 min at pH 10 and 20 °C.

The dye removal performance was further evaluated in relation to its dependence on the mass of adsorbent, OOP. As illustrated in Figure 9, the adsorption capacity demonstrates a gradual increase until an adsorbent concentration of 2.5 g L^−1^, at which point the adsorption of MB reaches its maximum (i.e., 90% or 428 mg g^−1^). This improvement can be attributed to the rise in adsorbent concentration within the solution, which results in an increase in binding sites available for dye adsorption, thereby enhancing the discoloration of the solution [89,90]. However, beyond the optimal adsorbent concentration, a marginal decrease in adsorption capacity is observed. This decline may be attributed to the previously described effect of the initial MB concentration, whereby a reduction in the driving force for mass transfer resulting from a decrease in the concentration gradient between the adsorbate and the adsorbent has been proposed. Moreover, additional phenomena may contribute to the observed decline in adsorption. For instance, aggregation of the adsorbent particles may reduce the available surface area with active sites, thereby decreasing the adsorption capacity [63,91].

**Figure 10:**
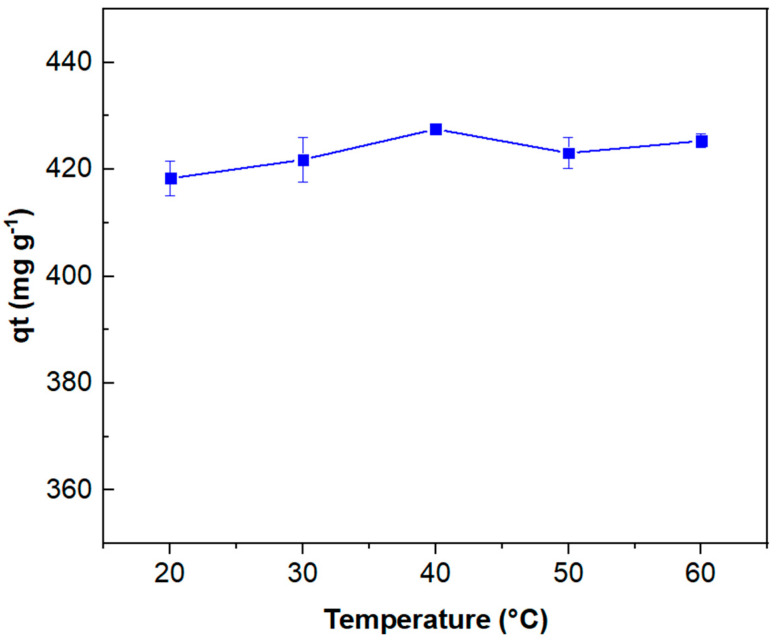
Effect of temperature on the adsorption efficiency of MB (460 mg L^−1^) by the OOP (2.5 g L^−1^), after stirring the samples for 200 min at pH 10.

The final parameter to be evaluated was the temperature, which systematically varied from 20 °C to 60 °C (Figure 10). The data indicates a modest increase (of approximately 2%) in the decolorization efficiency when the temperature was increased from 20 °C up to 40 °C. In these conditions, a decolorization yield of 93% is obtained, which corresponds to a dye removal of 428 mg g^−1^. Further increases in the temperature do not result in significant alterations to the dye adsorption capacity. The slight overall increase in the adsorbed amount with increasing temperature may, in part, be justified by the endothermic nature of the adsorption process, as previously discussed in references [90,92]. Furthermore, the thermal effect on the adsorbent porosity may potentially contribute to an enhanced adsorption capacity [93,94]. In any case, within the range studied, temperature has a minor effect on adsorption performance.

#### 3.2.4. Reusability of OOP

**Figure 11:**
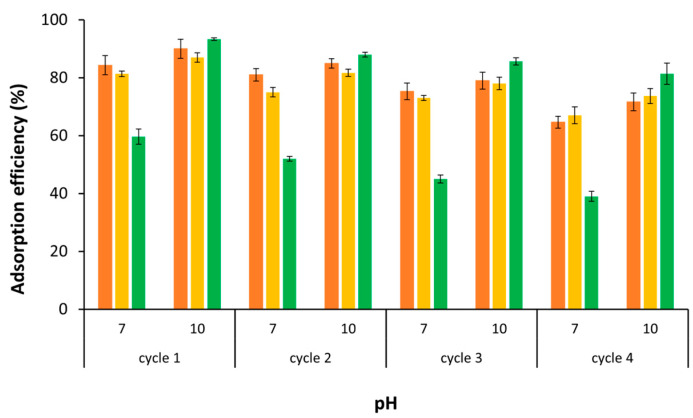
OOP performance in removing MB after consecutive adsorption and desorption cycles for both pH 7 and 10, at different dye concentrations of 100 (orange), 200 (yellow), and 460 (green) mg L^−1^.

The principal objective of the desorption studies is to elucidate the effective recyclability of the pomace-based adsorbents. The capacity to reuse the adsorbent through multiple sorption/desorption cycles represents a substantial advantage, contributing to both economic feasibility and practical applicability and sustainability. In this study, ethanol was selected as the solvent to drive the MB desorption from the OOP, thereby greatly enabling its reuse in a sustainable and cost-effective manner. A series of four consecutive cycles of adsorption/desorption experiments were conducted in batch mode. Figure 11 illustrates the variations in MB adsorption performance as a function of dye concentration.

The data indicates a slight decline in the adsorption efficiency for the three examined dye concentrations during the successive adsorption and desorption cycles. Nevertheless, after four cycles, the adsorption efficiency remains remarkably above ca. 70%. It is probable that the minor losses can be attributed to a slow and gradual degradation of the adsorbent material upon reuse [95]. The results suggest that the pomace-based adsorbent can be recycled and reused for several cycles while maintaining a high level of MB extraction capacity.

Although direct comparisons with existing literature are often not straightforward, it is possible to see that the OOP-based adsorbent developed in this work is notable for its high maximum adsorption capacity of 428 mg g^−1^ and removal efficiency of over 93% when compared with some other bio-based adsorbents found in the literature (see Table 5). The OOP biosorbent is both environmentally and economically advantageous and is notable for its simplicity of preparation, requiring only minimal quantities of chemicals and minimal energy consumption. These advantages render the pomace particularly well-suited to the biosorption of MB in liquid media, thereby promoting its use in sustainable and economically viable water treatment processes.

## 4. Conclusions

This study investigates the potential of defatted Moroccan OOP residues as a sustainable alternative for the adsorption of MB, a pervasive dye with detrimental environmental consequences. The material exhibited an aggregated and porous structure, displaying both amorphous and crystalline features, which are likely attributable to its lignocellulosic constituents. Additionally, various functional groups, including ketones, hydroxyls, carboxylic acids, and phenolics, were also identified, indicating the potential for diverse interactions between the bio-based adsorbent and the dye. The kinetic modeling and adsorption isotherm assays enabled the binding mechanism between MB and the pomace adsorbent to be elucidated. In particular, it was found that an equilibrium time of ca. 200 min is sufficient to remove the majority of the dye from aqueous media under the evaluated conditions, following a pseudo-second-order model. The optimization of the adsorption parameters suggests that the adsorption of MB is more favorable at an initial dye concentration of 100 mg L^−1^, pH 10, and an adsorbent dosage of 2.5 g. Furthermore, the process displays stability with increasing temperature, achieving a removal efficiency of 93% (428 mg g^−1^). The data additionally indicate that the adsorption process is occurring in a monolayer, which is consistent with the parameters derived from the Langmuir and BET isotherms. The parameters derived from the Langmuir, Freundlich, and D-R isotherms indicate that the adsorption process is favorable, with the binding of the dye to the pomace adsorbent most likely occurring through a compromise between electrostatic interactions, hydrogen bonding, and π-π interactions. Furthermore, the extracted thermodynamic parameters indicate that the adsorption of MB on OOP is an endothermic and spontaneous process within the explored temperature range. The adsorbent can be effectively recycled and reused for several consecutive cycles, thus demonstrating its advantageous characteristics. In conclusion, this study demonstrates that olive oil pomace, a significant byproduct of the Mediterranean region, can be effectively transformed into a sustainable bio-based material for the removal of significant pollutants, such as the MB dye, through appropriate treatment and defatting. This not only contributes to the valorization of these abundant residues but also paves the way for the development of novel, efficient, and more sustainable bio-based materials for the removal of pollutants in aqueous media.

## Figures and Tables

**Table 1:** Adsorption types according to *R_L_* values [54].

R_L_	0 < R_L_ < 1	R_L_ > 1	R_L_ = 1	R_L_ = 0
**Adsorption type**	Adsorption is favourable	Unfavourable adsorption	Linear adsorption	Irreversible adsorption

**Table 2:** Fitting parameters for the PFO and PSO adsorption kinetic models.

	Pseudo First Order				Pseudo Second Order				
Concentration (mg L^−1^)	R^2^	K_1_ (min^−1^)	qe_the_ (mg g^−1^)	qe_exp_ (mg g^−1^)	R^2^	K_2_ (g mg^−1^ min^−1^)	qe_the_ (mg g^−1^)	qe_exp_ (mg g^−1^)	h (mg g^−1^ min^−1^)
**5**	0.707	0.003	1.16	3.96	0.99	0.02	3.98	3.96	0.068
**10**	0.518	0.002	2.12	9.09	0.99	0.01	8.97	9.09	0.090
**20**	0.500	0.002	4.96	20.9	0.99	0.004	20.8	20.9	0.073
**50**	0.732	0.003	12.3	46.5	0.99	0.001	46.9	46.5	0.060
**100**	0.371	0.002	9.61	96.8	1.0	0.001	97.1	96.8	0.18
**200**	0.531	0.003	22.1	183	1.0	0.001	185	183	0.15
**460**	0.209	0.002	12.6	237	0.99	0.006	238	237	1.5

**Table 3:** Summary of the parameters extracted using the Langmuir, Freundlich, Dubinin Radushkevich, and BET isothermal modeling.

**Langmuir**	q_m_ (mg g^−1^)	K_L_ (L mg^−1^)	R^2^	
	269	0.053	0.960	
**Freundlich**	1/n	K_F_[(mg g^−1^)(L mg^−1^)^1/n^]	R^2^	
	0.386	32.4	0.832	
**D-R**	K_D-R_(mol^2^ kJ^−2^)	E(kJ mol^−1^)	R^2^	
	0.005	10.1	0.882	
**BET**	q m (mg g^-1^)	K_S_ (L mg^−1^)	K_L_ (L mg^−1^)	R^2^
	299	0.046	0	0.935

Note: pH was 7 and the temperature was 20 °C. The adsorbent mass was kept at 1 g L^−1^ while the MB concentration was varied from 5 to 460 mg L^−1^.

**Table 4:** Thermodynamic parameters related to the adsorption of MB on the OOP powder.

Temperature (°C)	Thermodynamic Parameters		
	ΔH° (J mol^−1^ K^−1^)	ΔS° (J mol^−1^)	ΔG° (J mol^−1^)
20	5496	36	−355
30			−548
40			−827
50			−1003
60			−1177

**Table 5:** MB adsorption capacity of OOP compared with other biobased materials in literature.

Adsorbent	Adsorption Capacity (mg g^−1^)	Reference
***Pergularia tomentosa*** **L. fruit**	152	[96]
**Wheat shells**	21.5	[31]
**Olive stone**	44.5	[35]
**Green tea waste**	68.3	[97]
***Labeo rohita*** **fish scale**	667	[98]
**OOP**	**428**	**(This work)**

## Data Availability

Data are contained within the article.
